# Very-low-calorie ketogenic diet vs hypocaloric balanced diet in the prevention of high-frequency episodic migraine: the EMIKETO randomized, controlled trial

**DOI:** 10.1186/s12967-023-04561-1

**Published:** 2023-10-04

**Authors:** Massimiliano Caprio, Eleonora Moriconi, Elisabetta Camajani, Alessandra Feraco, Vincenzo Marzolla, Laura Vitiello, Stefania Proietti, Andrea Armani, Stefania Gorini, Caterina Mammi, Gabriella Egeo, Cinzia Aurilia, Giulia Fiorentini, Carlo Tomino, Piero Barbanti

**Affiliations:** 1 Laboratory of Cardiovascular Endocrinology, IRCCS San Raffaele, Rome, Italy; 2 Department of Human Sciences and Promotion of the Quality of Life, San Raffaele Roma Open University, Rome, Italy; 3 Laboratory of Flow Cytometry, IRCCS San Raffaele, Rome, Italy; 4 Clinical and Molecular Epidemiology, IRCCS San Raffaele, Rome, Italy; 5 Headache and Pain Unit, IRCCS San Raffaele, Rome, Italy; 6 Scientific Direction, IRCSS San Raffaele, Rome, Italy

**Keywords:** VLCKD, Migraine treatment, Prevention, Diet, Weight loss, Ketone bodies, Inflammatory state, Aldosterone, Pain, Rehabilitation

## Abstract

**Background:**

Migraine is the second world’s cause of disability. Among non-pharmacological treatments, nutritional intervention, particularly ketogenic diet, represents one of the most promising approaches.

**Methods:**

This a prospective, single center, randomized, controlled study aimed at evaluating the efficacy of a very low-calorie ketogenic diet (VLCKD) compared to a hypocaloric balanced diet (HBD) in migraine prophylaxis in patients affected by high-frequency episodic migraine (HFEM) with a Body Mass Index (BMI) > 27 kg/m^2^. Fifty-seven patients were randomly assigned to a VLCKD (group 1) or HBD (group 2). Group 1 patients followed a VLCKD for 8 weeks, followed by a low calorie diet (LCD, weeks 9–12), and a HBD (weeks 13–24), whereas group 2 patients followed a HBD from week 0 to 24. Anthropometric indexes, urine and blood chemistry were assessed at enrollment, baseline, weeks 4, 8, 12, and 24. Migraine characteristics were evaluated at baseline, weeks 8, 12 and 24. Change in monthly migraine days (MMDs) at weeks 5–8 compared to baseline was the primary endpoint. Secondary endpoints encompassed changes in visual analogue scale (VAS), Headache Impact Test-6 (HIT-6) and Short Form Health Survey-36 (SF-36) scores. We also studied effects on circulating lymphocytes and markers of inflammation, changes in plasma aldosterone and renin levels before and after VLCKD or HBD treatment.

**Results:**

Reduction from baseline in MMDs was greater in VLCKD compared to HBD group at week 8 (p = 0.008), at week 12 (p = 0.007), when ketosis had been interrupted by carbohydrates reintroduction, and at week 24 (p = 0.042), when all patients were following the same dietary regimen. Quality of life scores (SF-36) were improved in VLCKD group at week 8 and 12, and were also improved in HBD group, but only at week 12. Weight-loss was significantly higher in VLCKD group at week 8 (p = 0.002) and week 12 (p = 0.020). At the end of the study weight loss was maintained in VLCKD group whereas a slight weight regain was observed in HBD group. Inflammatory indexes, namely C reactive protein (CRP), neutrophil to lymphocyte ratio (NLR) and total white blood cell count (WBC) were significantly reduced (p < 0.05) in VLCKD group at week 12. Aldosterone plasma level were significantly increased in both groups at week 8, particularly in VLCKD group. However, electrolytes and renin plasma levels were never altered throughout the study in both groups.

**Conclusions:**

VLCKD is more effective than HBD in reducing MMD in patients with HFEM and represents an effective prophylaxis in patients with overweight/obesity.

*Trial registration* ClinicalTrials.gov identifier: NCT04360148.

## Background

Migraine is a neurological disorder characterized by recurrent episodes of headache associated with vegetative symptoms and represents the second leading cause of disability worldwide [1]. The last decades have witnessed an unprecedented number of studies which contributed to a significant increase in the understanding of migraine pathophysiology. The migraine attack results from the activation of the trigemino-vascular system caused by a hyperexcitable and hypometabolic brain which is sensitive to otherwise innocuous internal (body) or external (environment) stimuli [2]. Migraine is defined episodic (EM) when headaches occur on < 15 days/month and chronic (CM) when headaches are presents on > 15 days/month for > 3 months, with migraine features on > 8 days/month [3, 4]. EM patients progress to CM with a rate of 2.5% per year [5]. Notably, the risk of transition to CM is much higher (OR = 4.3; 95% CI 2.7–6.7) in subjects with high-frequency EM (HFEM: 8–14 days/month) compared to those with less than 5 headache days/month [6].

Many factors may contribute to migraine chronicization [7]. Among them, advanced age, head trauma, hormonal imbalances, lower socioeconomic status, smoking, analgesic overuse, substance abuse, stress, sleep disorders, metabolic disorders (obesity, diabetes, dyslipidemia, hypertension), neurological and autoimmune diseases and pro-inflammatory or pro-thrombotic states [8].

In particular, obesity is strongly linked to migraine, especially in women in the reproductive age [9]. For this reason, the role of lifestyle modifications has become increasingly important in order to reduce frequency and severity of migraine attacks [10] and improve quality of life [11]. Notably, a negative correlation between Body Mass Index (BMI) and responsiveness to monoclonal antibodies targeting the calcitonin-gene related peptide, the first selective and specific preventative migraine agents, has been documented [12].

The acronym SEED (Sleep, Exercise, Eat, and Diary) underlines that lifestyle change is required to reduce migraine burden [10]. In detail, a regular sleep, an aerobic exercise program (150–300 min of moderate-intensity aerobic exercise per week), a restricted calorie diet (in some cases elimination diets), an adequate hydration and, finally, a daily headache diary, can help the management of migraine attacks [13].

Among non-pharmacological approaches, nutritional intervention represents one of the most promising migraine treatment [14]. Calorie restriction is often recommended for people suffering from migraine, however, several specific nutritional strategies (gluten-free diet for celiac disease, histamine-free or tyramine-free diet, low-fat diets or low-glycemic index diets) have been used to improve symptoms of migraine [15–17].

Ketogenic diets (KDs) are high-fat, adequate-protein, very low carbohydrate diets which have been primarily used to treat refractory epilepsy in children since the 1920s [18]. Over the past 15 years, the interest in the KD dramatically increased providing therapeutic benefits to a wide range of different neurological conditions (Alzheimer’s disease, Parkinson’s disease (PD), migraine and depression) [19]. KD is a nutritional regimen based on the drastic reduction in carbohydrate intake (usually < 30–50 g/day) associated to a relative increase in protein and fat proportion. In this scenario, very low-calorie ketogenic diet (VLCKD) represents a calorie-restricted nutritional protocol (600–800 kcal/day), limited in time [14], that has become popular to promote a rapid and efficient weight loss in patients with obesity [20], determining a rapid improvement of chronic inflammation, insulin resistance and metabolic disorders. This nutritional protocol has proven efficacy also in migraine [21].

The potential favorable effects of VLCKD on migraine include a reduction of brain cortical excitability through the activation of astrocyte metabolism which favors glutamate conversion to glutamine and its conversion to GABA [21]. Furthermore, VLCKD reduces the propagation of cortical spreading depression, the neurophysiological event underpinning migraine aura [21, 22], prevents neuroinflammation and reduces mitochondrial reactive oxygen species (ROS) production [21]. A further possible mechanism of action involved in the efficacy of VLCKD in migraine is linked to a protective role of ketones on impaired intracerebral glucose metabolism [14]. Importantly, obesity worsen the clinical phenotype of migraine, favoring its chronicization [23]**.** The mechanisms involved are still not well understood. Increased level of calcitonin gene-related peptide (CGRP), a postsynaptic mediator of the migraine trigeminovascular inflammation, has been found in patients with obesity, particularly in women [24]. Importantly, obesity is characterized by a chronic, systemic low-grade state of inflammation, directly contributing to neurovascular inflammation and sustaining the pathophysiology of migraine [25].

The Renin–Angiotensin–Aldosterone System (RAAS) has a major role in the regulation of blood pressure, hydrosaline metabolism, autonomic pathways and neuroendocrine systems, and its excessive activation is observed in obesity, hypertension and heart failure [26, 27]. Importantly, it is clear since decades that RAAS activation plays a relevant role in the pathophysiology of migraine [28], probably due to its role on cerebrovascular flow, opioid metabolism and inflammation. For this reason lipophilic ACE inhibitors and Angiotensin Receptor Blockers are used as migraine prophylactic agents [29]. However, the exact mechanisms through which pharmacological RAAS blockade has a therapeutic role is still unclear.

This randomized placebo-controlled study is aimed to compare the efficacy of VLCKD vs HBD in migraine prevention in patients with overweight/obesity affected by high-frequency episodic migraine (HFEM: 8–14 days/month).

## Materials and methods

The trial protocol, consistent with the principles set out in the Declaration of Helsinki and written according to the Good Clinical Practice, was approved by the IRCSS San Raffaele Ethics Committee on January 22, 2020. All patients provided written informed consent. The study has been registered with clinicaltrials.gov (https://clinicaltrials.gov/show/NCT04360148) ClinicalTrials.gov identifier: NCT04360148. This trial is a single center, randomized, longitudinal controlled trial of a nutritional intervention conducted between March 2020 and December 2021. It was based on a predefined randomization list and the allocation by the two group was generated by using an electronic case report form (eCRF).

### Study population

We considered all consecutive patients affected by HFEM aged 18–65 years with a Body Mass Index (BMI) ranging from 27 kg/m^2^ to 35 kg/m^2^ [30] visited at the Headache and Pain Unit of IRCCS San Raffaele Roma. After signing the informed consent, all patients were evaluated by specifically trained neurologists who gathered detailed information of sociodemographic and clinical characteristics of migraine using a semi structured questionnaire [31]. Each patient was also evaluated by a psychologist, in order to prevent the risk of a poor adherence to the assigned nutritional regimen; any time during the trial every patient had the opportunity to meet the psychologist if any difficulties occurred. Patients were required to have discontinued any preventive migraine treatment from at least 3 months before the screening visit. Inclusion and exclusion criteria are summarized in Table 1.

**Table 1:** Inclusion and exclusion criteria

Inclusion criteria	Exclusion criteria
27 kg/m^2^ < BMI < 35 kg/m^2^	BMI < 27 or > 35 kg/m^2^
Diagnosis of HFEM	Medication overuse, or any other primary or secondary headache [28]
Signing of the informed consent	Psychiatric disorders or any other condition or disease influencing treatment adherence
Migraine onset < 50 years	Type I diabetes mellitus or type II diabetes mellitus treated with insulin
Preventive migraine treatment discontinuation since at least 3 months (including RAAS inhibitors)	Use of antidepressants, anticonvulsants, lithium carbonate or neuroleptics for psychiatric comorbidities
Agreement to follow all study procedures, including follow-up visits	Use of potassium-sparing diuretics and RAAS inhibitors
Negative pregnancy test, performed on urine sample	Use of neurostimulators for migraine
Use of a valid contraceptive method throughout the study	Intake of supplements affecting weight
Agreement for all study participants not to divulgate study information	Intake of sugar-containing supplements
	Pregnancy or breastfeeding
	Alcohol abuse
	Other neurological, cardiovascular, liver, respiratory, hematologic, autoimmune diseases issues that could, in the opinion of the investigator, influence the study results

### Dietary intervention

All the eligible subjects were randomly allocated to the VLCKD (treatment) or HBD (active comparator) group. Each nutritional program lasted 24 weeks. VLCKD is a restricted multiple-steps dietary regimen including nutritional supplements and replacement meals. In the first step (four weeks), four or five replacement meals per day, according to patients’ specific nutritional needs, were used. In the second step (four weeks), one and subsequently two replacement meals were replaced with conventional food containing proteins (meat, fish, eggs, soy) at lunch and/or dinner. During the first two steps, diet provided a minimum protein content based on a Population Reference Intake for protein adjusted for people with overweight and obesity (75–105 g/day), carbohydrate intake was drastically restricted (30–50 g/day) and lipid intake was very low and mostly derived from olive oil (20 g per day, linoleic acid 11 g/day, alpha-linoleic acid 1.4 g/day), according to the European Food Safety Authority (EFSA) [32]. The amount of daily fiber intake was approximately 25 g/day, as requested from Italian Guidelines (LARN 2014), mostly deriving from vegetable servings with low glycaemic index. Total energy intake was < 800 kcal/day. Recommended water intake was at least 2.5 lt/day. To avoid micronutrient deficiencies, mineral and vitamin supplements were recommended and only erythritol or steviol glycosides were allowed as sweeteners [33]. The VLCKD diets were prepared by New Penta s.r.l. (Cuneo, Italy) and were delivered in preassembled boxes.

In the following four weeks (LCD phase), carbohydrates were gradually reintroduced, starting from foods with the lowest glycemic index (fruit, dairy products), followed by foods with moderate and high glycemic index (legumes, bread, pasta and cereals). The goal was to achieve a hypocaloric balanced diet (HBD), that was the same of the control group. From week 12 week to week 24 all subjects enrolled continued the study following HBD.

The HBD group followed a hypocaloric balanced diet for 24 weeks. Total daily average energy intake was 1500–1600 kcal/day and macronutrients composition was based on the Mediterranean Diet [lipid: 30% of total daily energy (10% MUFA, 10% PUFA, 10% SFA); carbohydrates: 55% of total daily intake; daily protein intake was approximately 0.8–1.5 g/kg of ideal body weight].

**Fig. 1:**
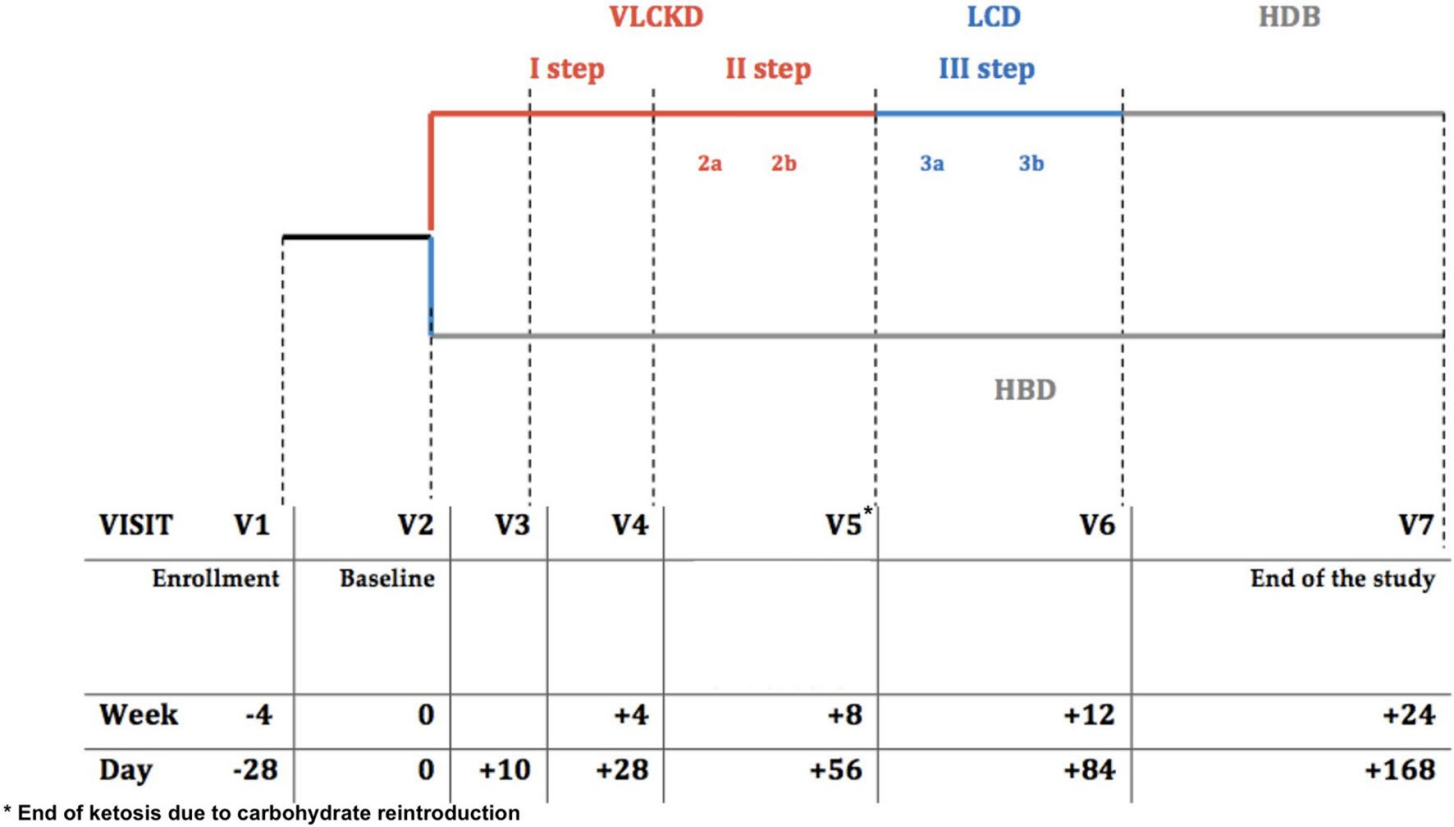
Emiketo: visits’ timeline

The summary of timeline of visits is shown in Fig. 1.

### Anthropometric assessment and blood exams

Periodically, participants were monitored through physical examination (i.e. anthropometric measurements, blood pressure, heart rate) and blood exams.

Body weight (BW), height, systolic and diastolic blood pressure (BP), waist circumference (WC) and hip circumference (HC) were measured at V1 (enrollment visit), V2 (baseline), after 10 days (V3) from starting dietary protocol and every 4 weeks (V4-V5-V6-V7). Anthropometric data were recorded after an overnight fast under resting conditions.

BW (kg) was measured to the nearest 0.01 kg, using an accurate balance scale (Invernizzi, Rome, Italy).

Height was rounded to the closest 0.5 cm. BMI was calculated as weight divided by squared height in meters (kg/m^2^). WC was measured midway between the costal arch and the iliac crest; HC was measured at the symphysis-greater trochanter level to the closest 1 cm. Systolic and diastolic BP were measured using a mercury-gravity manometer.

Fasting blood was collected into ethylenediaminetetraacetic acid (EDTA) at 3000 × g for 10 min. Plasma was separated and stored at – 80 °C for hormonal analysis (aldosterone and renin evaluation).

Fasting glycemia, lipid profile, electrolytes, liver enzymes, and renal function parameters were measured.

Ketosis was confirmed by ß-Hydroxybutyrate capillary blood detection by using a portable meter (Wellion Galileo ketone test strips). The threshold value for nutritional ketosis was set at 0.5 mmol/L of ß-Hydroxybutyrate [34].

The inflammatory profile was analysed throught measurement of C-reactive protein plasma level, neutrophil to lymphocyte ratio (NLR) and platelet to lymphocyte ratio (PLR). Finally, the effects of nutritional regimens upon RAAS activation were evaluated through measurement of aldosterone and direct renin in the plasma.

### Flow cytometric analysis of peripheral blood lymphocytes

To evaluate the relative proportions of lymphocyte populations in peripheral blood, the following panel of fluorochrome conjugated antibodies was used: anti CD45 BUV 395, anti CD3 APC R700, anti CD19 APC, anti CD16 PE-Cy7, anti CD56 BB515, anti CD4 BUV 7373, anti CD8 BV 785, anti CD25 BV 421, anti CD127 BB700, anti CD45RA BV605, anti CD197 BV711, anti FoxP3 PE, anti RORγT BV650 (all from BD Biosciences, Milan, Italy). Blood samples were stained with anti membrane associated molecules and, after incubation, erythrocytes were lysed using FACS lysing solution, according to manufacturer’s instruction. To analyze the expression of transcription factors (FoxP3 and RORγT), samples were fixed and permeabilized using Transcription Facytors Buffer Set (BD Bioscience), according to standard protocol. Samples were acquired and analyzed on a LSR Fortessa X-20 (Becton Dickinson), using FACS DIVA v8.0.2 software.

### Questionnaires

At baseline and during the entire study duration, patients were asked to fill-out a paper–pencil diary recording monthly migraine days (MMDs), monthly analgesic intake (n/month), and rating pain intensity (0–10, Visual Analog Scale, VAS) [35]. Pain disability was measured using the Headache Impact Test (HIT-6) [36] and Migraine Disability Assessment Scale (MIDAS) [37]. Quality of life was assessed through the Short Form Health Survey (SF-36) questionnaire. Vivacity, agitation, sadness, calmness, energy, discouragement, happiness, and satiety were evaluated using a 5-point scale [38].

The eight domains of SF-36 may be grouped into two macro -areas: a physical dimension, represented by the Physical Component Summary (PCS), and a mental dimension, represented by the Mental Component Summary (MCS). All scales do contribute in different proportions to the scoring of both PCS and MCS measures [39].

Adherence to the dietary interventions was evaluated through a non-validated test, while before starting study, a daily food diary was required to be filled out in order to study eating habits.

### Outcomes of the study

The primary endpoint was the change in MMDs at weeks 5–8 compared to baseline. Secondary endpoints encompassed change in MMDs at weeks 9–12 and 21–24 compared to baseline, and change in monthly analgesic intake, VAS, HIT-6 and SF-36 scores at weeks 5–8, 9–12 and 21–24 compared to baseline. Other secondary enpoints were changes in antropometric data, durability of weight loss maintenance, effects on circulating lymphocytes and inflammatory markers, and changes in plasma aldosterone and renin levels before and after VLCKD or HBD treatment at weeks 8 and 12.

### Statistical analysis

Statistical data were reported as mean ± standard deviation (SD) for continuous variables and frequency and percentage for categorical variables. Quantitative variables were studied using analytical method in order to test if data were normally distributed (Kolmogorov–Smirnov/Shapiro–Wilk test). Chi square test was used to compare frequency between categorical variables. Fisher test was applied when expected frequency was < 5. For comparisons between groups (VLCKD and HBD) t-test for independent samples or the Mann–Whitney test were used if they did not respect normality; to evaluate the pre-post treatment within the group, t-test was used for paired samples or the Wilcoxon test if they did not respect the normality. Due to the exploratory nature of the study, no correction for multiple comparisons was applied. Given the high dropout rate, especially in the control group, a sensitivity analysis was performed to evaluate the presence of attrition bias.

The level of significance was set at p < 0.05. Statistical analyses were performed using the Statistical Package for Social Sciences (SPSS) software (version 28, IBM, Armonk, New York, NY, USA).

## Results

Fifty-seven patients were enrolled in the study and randomly assigned to VLCKD (n = 29) or HBD (n = 28). Four patients (13.8%) discontinued the study in VLCKD group, whereas fourteen patients (50%) dropped out in HBD group. We explored potential deviations as attrition bias using a sensitivity analysis, and attrition bias was not observed. In fact, when comparing the group that completed the study and the drop-out group, no statistically significant differences emerged, except for the SF36 RP.

### Baseline characteristics of the study groups (V2, week 0)

At baseline, there were no differences in gender balance among groups (96.7% female in VLCKD group and 93.1% in HBD group), age (p = 0.361) or anthropometric parameters (BMI p = 0.051, WC p = 0.112), except for HC, which was significantly higher in VLCKD group (p = 0.029).

Diastolic and systolic blood pressure, as well as biochemical parameters were similar between two groups. Migraine frequency (MMDs) was higher in VLCKD group than HBD group (p = 0.007). Ten patients also referred aura episodes: 4 were allocated in the VLCKD group and 6 in HBD group. At study baseline, all patients were taking analgesics for acute migraine attacks; no significant differences in monthly analgesic intake was observed. Table 2 reports baseline patients’ characteristics.

**Table 2:** Baseline characteristics of participants enrolled in VLCKD and HBD groups

	ALL	VLCKD	HBD	p-value
Partecipants (n)	N = 59	N = 30	N = 29	–
Gender, Female, n (%)	56 (94.9)	29 (96.7)	27 (93.1)	0.612
Age (years)	42.6 ± 11.5	41 ± 12.3	44 ± 10.5	0.361
Class of age (n)				
18–35	19 (32.2)	12 (40.0)	7 (24.2)	
36–50	21 (35.6)	10 (33.3)	11 (37.9)	
51 +	19 (32.2)	8 (26.7)	11 (37.9)	0.402
Weight (kg)	88.6 ± 16.6	91.8 ± 16.0	85.3 ± 16.9	0.143
BMI, median (IQR)	31.6 (27.8–35.2)	33.65 (29.3–36.0)	29.0 (27.5–34.1)	0.051
WC (cm)	95.7 ± 12.6	98.3 ± 12.3	93.0 ± 12.4	0.112
HC (cm), median (IQR)	115.0 (109.0–124.0)	120.0 (110–126)	112.0 (106.2–119.2)	0.029
WHR	0.8 ± 0.8	0.8 ± 0.07	0.8 ± 0.09	0.960
Systolic BP (mmHg)	122.0 ± 14.0	126.1 ± 12.5	117.8 ± 14.5	0.026
Diastolic BP (mmHg)	81.3 ± 9.4	83.3 ± 9.0	79.5 ± 9.5	0.128
Heart frequency	76.4 ± 9.1	75.8 ± 8.8	77.2 ± 9.4	0.581
Comorbidity (n)				
≥ 1	49 (83.1)	23 (76.7)	26 (89.7)	0.108
Comorbidity (n)				
Cardiovascular	5 (17.2)	–	5 (17.2)	0.052
Hypertension	14 (24.1)	8 (27.6)	6 (20.7)	0.539
Metabolic	24 (41.4)	10 (34.5)	14 (48.3)	0.286
Gastrointestinal	24 (40.7)	11 (36.7)	13 (44.8)	0.524
Respiratory	5 (8.5)	3 (10.0)	2 (6.9)	1.000
ENT	1 (1.7)	–	1 (3.4)	0.492
Urologic	3 (5.1)	1 (3.3)	2 (6.9)	0.612
Rheumatologic	1 (1.7)	–	1 (3.4)	0.492
Hematologic	2 (3.4)	2 (6.7)	–	0.492
Neoplastic	1 (1.7)	–	1 (3.4)	0.492
Gynecological	12 (20.3)	6 (20.0)	6 (20.7)	0.948
Orthopedic	4 (6.8)	1 (3.3)	3 (10.3)	0.353
TMJ dysfunction	1 (1.7)	1 (3.3)	–	1.000
Trauma	4 (6.9)	1 (3.3)	3 (10.7)	0.344
Psychiatric	10 (17.2)	5 (16.9)	5 (17.9)	0.905
Age migraine onset, mean ± sd	18.6 ± 9.3	19.5 ± 7.8	17.7 ± 10.8	0.462
Smoking, n (%)	15 (25.4)	6 (20.0)	9 (31.0)	0.330
Sport activity, n (%)	19 (32.2)	9 (30.0)	10 (34.59	0.713
Alcohol occasional consumption, n (%)	31 (54.4)	17 (60.7)	14 (48.3)	0.346
Previous pregnancies, n (%)	25 (44.6)	10 (34.5)	15 (55.6)	0.113
Menopause, n (%)	15 (27.3)	7 (24.1)	8 (30.8)	0.581
E/P therapy, n (%)	5 (8.9)	4 (13.8)	1 (3.7)	0.185
Quality of sleep, n (%)				
Poor	9 (26.5)	6 (31.6)	3 (20.0)	
Good	25 (73.5)	13 (68.4)	12 (80.0)	0.697
Aura, n (%)	10 (17.2)	4 (13.8)	6 (20.7)	0.730
Aura duration (minutes)	26.1 ± 21.4	23.7 ± 24.3	28.0 ± 21.7	0.789
MMDs (n/month)	13.2 ± 5.3	15.0 ± 5.6	11.3 ± 4.2	0.007
Monthly analgesic intake (n/month, %)	56 (96.6)	29 (100.0)	27 (93.1)	0.491

BMI, body mass index; WC, waist circumference; HC, hip circumference; BP, blood pressure; ENT, Ear, nose and throat, TMJ, temporomandibular joint; E/P, estrogen/progestins; MMDs, monthly migraine days

All values are presented as mean ± standard deviation or median (interquartile range, IQR). Differences were considered statistically significant when *p* was < 0.05. Significant *p* values are highlighted in bold

At baseline, no difference in pain intensity, disability and quality of life scores emerged between the two groups (Table 3).

**Table 3:** MIDAS, HIT-6, SF-36 and VAS scores at baseline

	ALL	VLCKD	LCD	p-value
MIDAS	55.0 (31.0–85.0)	54.0 (33.0–98.5)	55.0 (21.0–82.0)	0.814
HIT-6	66.0 (64.0–68.0)	66.0 (64.0–68.0)	66.0 (62.0–68.0)	0.332
SF-36 total score	103.8 ± 5.3	103.1 ± 5.4	104.5 ± 5.1	0.330
• SF-36 PF	75.0 (60.0–90.0)	70.0 (55.0–82.5)	85.0 (70.0–95.0)	0.063
• SF-36 RP	25.0 (0.0–75.0)	50.0 (0.0–75.0)	25.0 (0.0–50.0)	0.515
• SF-36 BP	71.0 (62.0–71.0)	71.0 (62.0–71.0)	71.0 (62.0–71.0)	0.554
• SF-36 GH	53.0 ± 15.3	52.2 ± 15.7	53.8 ± 15.2	0.690
• SF-36 VT	45.0 (30.0–50.0)	40.0 (30.0–45.0)	45.0 (25.0–55.0)	0.741
• SF-36 SF	55.0 (38.0–63.0)	55.0 (38.0–63.0)	55.0 (38.0–63.0)	0.890
• SF-36 RE	33.0 (0.0–67.0)	67.0 (0.0–83.5)	33.0 (0.0–33.0)	0.107
• SF-36 MH	58.2 ± 14.2	57.8 ± 15.1	58.7 ± 13.5	0.809
VAS	8.0 (7.0–9.0)	8.0 (7.0–9.0)	8.0 (7.0–9.0)	0.552

MIDAS, Migraine Disability Assessment Scale; HIT-6, Headache Impact Test; SF-36, Short Form Health Survey

SF-36 PF, physical functioning; SF-36 RP, role physical; SF-36 BP, bodily pain; SF-36 GH, general health; SF-36 VT, vitality; SF-36 SF, social functioning; SF-36 RE, role emotional; SF-36 MH, mental health. All values are presented as median (interquartile range, IQR) or mean ± standard deviation

### Comparative results (week 8 vs week 0 and week 12 vs week 0)

#### Migraine frequency and severity

At week 8, patients on VLCKD showed a statistcally significant greater reduction in MMDs compared to HBD (− 6.4 ± 4.8 vs − 2.2 ± 5.0, p = 0.008). Importantly, MMDs reduction remained significantly higher in patients allocated to the VLCKD group also after stopping ketosis by carbs reintroduction (week 12: − 7.2 ± 5.42 vs − 3.13 ± 3.58, p = 0.007), and when patients were shifted to HBD (week 24: − 6.8 ± 6.42 vs − 3.6 ± 3.3, p = 0.042) (Tables 4 and 5).

**Table 4:** Comparison of anthropometric data and questionnaires scores between week 8 and week 0 (data expressed as ∆)

	ALL	VLCKD	HBD	p-value
	46	28 (60.9)	18 (39.1)	
Weight (kg)	− 6.7 ± 4.4	− 8.2 ± 4.5	− 4.3 ± 2.9	0.002
BMI (kg/m^2^)	− 2.4 ± 1.6	− 3.0 ± 1.6	− 1.5 ± 1.1	0.002
WC (cm)	− 6.4 ± 6.0	− 8.0 ± 6.5	− 3.9 ± 4.2	0.021
HC (cm)	− 6.2 ± 4.9	− 7.7 ± 5.0	− 3.8 ± 3.7	0.006
Systolic BP (mmHg)	− 5.9 ± 14.4	− 10.8 ± 15.7* p < 0.001	1.73 ± 9.52	0.008
Diastolic BP (mmHg)	− 3.5 ± 11.6	− 7.04 ± 10.3* p = 0.01	− 0.8 ± 12.2	0.097
Heart Frequency	0.09 ± 9.0	− 0.61 ± 10.37	1.11 ± 6.71	0.538
MMDs (n/month)	− 4.7 ± 5.2	− 6.4 ± 4.8	− 2.2 ± 5.0	0.008
HIT-6	− 5.3 ± 6.5	− 5.2 ± 6.3	− 5.4 ± 6.9	0.919
VAS	− 1.8 ± 2.4	− 1.7 ± 1.7	− 1.8 ± 2.4	0.773
SF-36 PF	11.18 ± 40.8	10.6 ± 18.55* p = 0.009	− 0.59 ± 22.07	0.083
SF-36 RP	− 5.70 ± 95.9	9.0 ± 41.38	14.7 ± 54.5	0.702
SF-36 BP	− 4.0 ± 30.13	3.48 ± 9.9	7.5 ± 20.0	0.397
SF-36 GH	− 2.67 ± 29.0	5.68 ± 11.7* p = 0.023	8.4 ± 17.4	0.555
SF-36 VT	3.12 ± 41.2	9.6 ± 22.7* p = 0.045	6.5 ± 15.8	0.625
SF-36 SF	0.96 ± 52.9	12.2 ± 22.7* p = 0.013	11.2 ± 30.24	0.907
SF-36 RE	7.90 ± 115.5	11.9 ± 62.3	4.06 ± 47.0	0.660
SF-36 MH	2.45 ± 29.9	5.28 ± 15.0	2.82 ± 14.2	0.598
SF-36 total	3.67 ± 14.3	2.30 ± 7.6	− 1.37 ± 6.20	0.118

BMI, body mass index; WC, waist circumference; HC, hip circumference; BP, blood pressure; MMDs, monthly migraine days; MIDAS, Migraine Disability Assessment Scale; HIT-6, Headache Impact Test; VAS, Visual Analogue Scale; SF-36, Short Form Health Survey; SF-36 PF, physical functioning; SF-36 RP, role—physical; SF-36 BP, bodily pain; SF-36 GH, general health; SF-36 VT, vitality; SF-36 SF, social functioning; SF-36 RE, role—emotional; SF-36 MH, mental health

We highlighted with * significant differences between baseline and week 8 in each group

All values are presented as mean ± standard deviation, or percentage. Differences were considered statistically significant when *p* was < 0.05. Significant *p* values are highlighted in bold

**Table 5:** Comparison of anthropometric data and questionnaires scores between week 12 and week 0 data (expressed as ∆)

	ALL	VLCKD	HBD	p-value
	41 (100)	26 (63.4)	15 (36.6)	
Weight (kg)	− 7.5 ± 5.7	− 9.1 ± 6.4	− 4.9 ± 2.7	0.020
BMI (kg/m2)	− 2.7 ± 2.0	− 3.3 ± 2.2	− 1.8 ± 1.0	0.016
WC (cm)	− 7.5 ± 7.0	− 9.2 ± 7.4	− 4.6 ± 5.2	0.041
HC (cm)	− 7.4 ± 5.5	− 8.8 ± 5.9	− 4.9 ± 3.9	0.029
Systolic BP (mmHg)	− 8.5 ± 16.2	− 11.3 ± 16.6* p = 0.005	− 5.2 ± 15.0	0.259
Diastolic BP (mmHg)	− 4.5 ± 10.2	− 5.58 ± 9.57* p = 0.031	− 4.2 ± 10.3	0.673
Heart Frequency	1.3 ± 10	2.48 ± 10.1	− 0.6 ± 9.76	0.352
MMDs (n/month)	− 5.7 ± 5.2	− 7.2 ± 5.4	− 3.1 ± 3.6	0.014
Migraine attack duration (hours)	− 13.5 ± 20.0	− 13.8 ± 19.3	− 12.9 ± 22.2	0.911
MIDAS	− 25.9 ± 38.4	− 22.0 ± 43.3	− 32.5 ± 28.3	0.426
HIT-6	− 6.0 ± 6.9	− 6.3 ± 6.7	− 5.4 ± 7.5	0.697
VAS	− 1.9 ± 1.9	− 1.8 ± 1.6	− 2.1 ± 2.3	0.632
SF-36 PF	12.3 ± 11.3	12.83 ± 10.6 ** p < 0.001	11.42 ± 12.77 * p = 0.005	0.722
SF-36 RP	23.6 ± 46.8	18.48 ± 47.80	32.14 ± 45.39 * p = 0.020	0.396
SF-36 BP	12.0 ± 15.5	12.30 ± 13.58 ** p < 0.001	11.57 ± 18.82 *p = 0.039	0.891
SF-36 GH	6.8 ± 12.5	6.65 ± 14.81 * p = 0.043	7.07 ± 7.95 * p = 0.005	0.923
SF-36 VT	13.9 ± 16.1	16.52 ± 18.05 ** p < 0.001	9.64 ± 11.84 *p = 0.009	0.214
SF-36 SF	14.7 ± 23.7	13.95 ± 24.76 *p = 0.013	16.0 ± 22.66 *p = 0.02	0.803
SF-36 RE	23.5 ± 55.5	20.26 ± 63.43	28.71 ± 41.07 *p = 0.021	0.660
SF-36 MH	6.8 ± 12.6	8.0 ± 14.16 **p = 0.0013	4.86 ± 9.57	0.469
SF-36 total	1.7 ± 6.3	0.95 ± 6.50	2.71 ± 6.05	0.426

BMI, body mass index; WC, waist circumference; HC, hip circumference; BP, blood pressure, physical functioning (PF), role—physical (RP), bodily pain (BP), general health (GH), vitality (VT), social functioning (SF), role—emotional (RE), and mental health (MH). We have highlighted with * the significant differences between baseline and week 12 in each group

All values are presented as mean ± standard deviation, or percentage. Differences were considered statistically significant when *p* was < 0.05. Significant *p* values are highlighted in bold

**Fig. 2:**
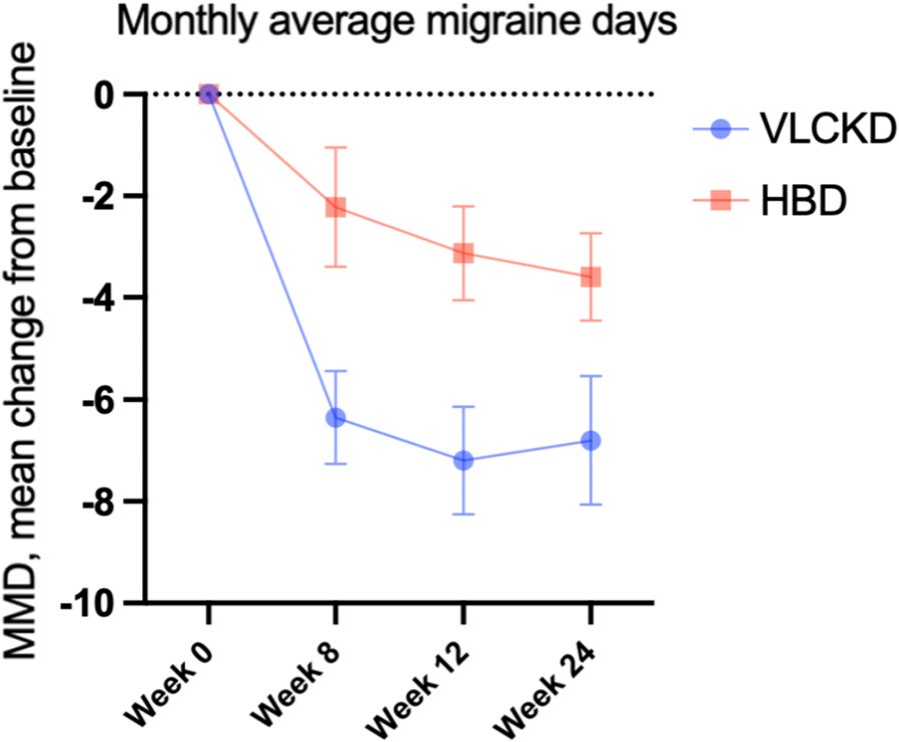
Variations in monthly migraine days (MMDs) in VLCKD and HBD groups at weeks 0, 8, 12 and 24 (V2, V5, V6 and V7)

Figure 2 shows the variations in MMDs in VLCKD and HBD groups during the study.

No significant differences were observed for migraine severity (p = 0.773; 0.632) between groups at week 8 and 12 when compared with week 0 (Tables 4 and 5).

#### Anthropometric measures

Weight-loss, reduction of BMI and of anthropometric measures (WC and HC) were significantly higher in the VLCKD group, as compared with the HBD group, at week 8 (− 8.2 ± 4.5 vs − 4.3 ± 2.9, p = 0.002; − 3.0 ± 1.6 vs − 1.5 ± 1.1, p = 0.002 − 8.0 ± 6.5 vs − 3.9 ± 4.2, p = 0.021; − 7.7 ± 5.0 vs − 3.8 ± 3.7, p = 0.00). These observations were also confirmed at week 12, when all patients in VLCKD group had already reintroduced conventional foods, whereas HBD group continued the same dietary pattern as before (− 9.1 ± 6.4 vs − 4.9 ± 2.7, p = 0.020; − 3.3 ± 2.2 vs − 1.8 ± 1.0, p = 0.016; − 9.2 ± 7.4 vs − 4.6 ± 5.2, p = 0.041; − 8.8 ± 5.9 vs − 4.9 ± 3.9, p = 0.029) (Tables 4 and 5). At the end of the study (week 24), mean weight loss remained unchanged in VLCKD group (− 9.1 ± 6.4) whereas a slight decrease was observed (− 4.3 ± 2.9) in HBD group, when compared with week 12. No significal differences of mean WC were observed in both groups at week 24, when compared with week 12.

**Fig. 3:**
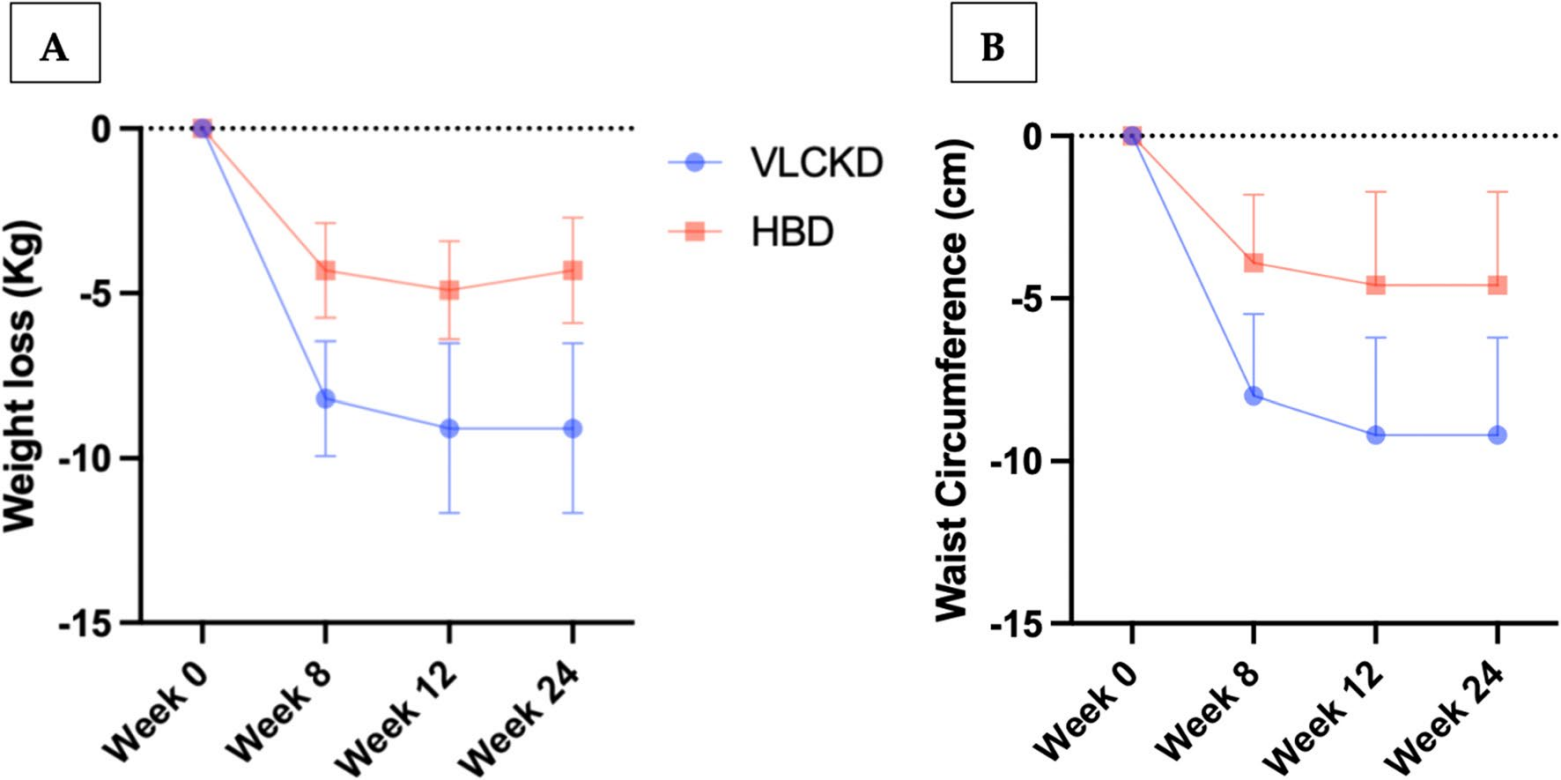
**A** Variations in mean weight loss in VLCKD and HBD groups at week 0, 8, 12 and 24. **B** Variations in waist circumference (cm) in VLCKD and HBD groups at week 0, 8, 12 and 24

Figure 3 shows the variations in mean weight loss (panel A) and waist circumference (panel B) in VLCKD and HBD groups during the study.

At week 8, systolic and diastolic blood pressure in VLCKD group were significantly reduced, as compared to week 0, whereas sistolic blood pressure in VLCKD group was significantly reduced as compared to HBD group (Table 4).

At week 12 systolic and diastolic blood pressure were still significantly reduced in VLCKD group when compared to baseline, whereas the comparison between two groups did not show any significant difference (Table 5).

No significal differences of mean heart frequency were observed in both groups at week 8 and 12, when compared with baseline (Tables 4 and 5).

#### Quality of life and adherence to diet

No significant differences were observed on scores of pain intensity or disability (MIDAS, HIT6 and VAS) between the two groups at week 8 (Table 4) and week 12 (Table 5).

At week 8, a significant improvement in scores of several domains of SF-36 was observed only in VLCKD group (physical functioning, PF p = 0.009, general health, GH p = 0.023, vitality, VT p = 0.045, social functioning, SF p = 0.013, Table 4), and not in HBD group. Moreover, at week 12 a general improvement of all area (physical and mental components) was observed either in VLCKD and HBD group (Table 5).

**Fig. 4:**
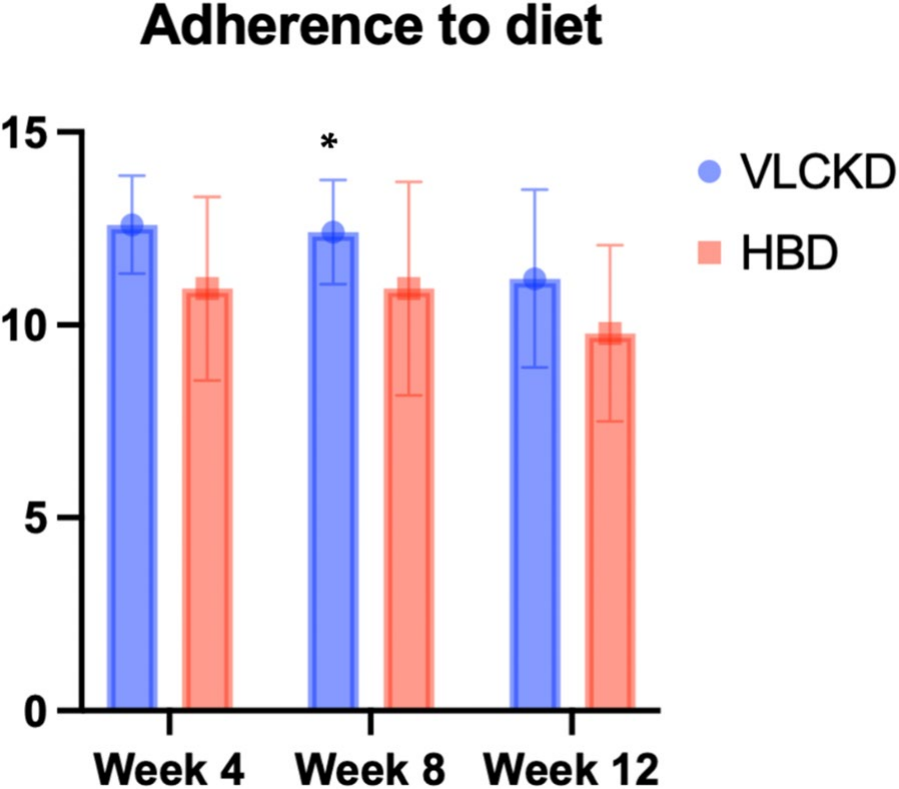
Adherence scores in VLCKD and HBD groups at weeks 4, 8 and 12

Finally, adherence to diet was higher in VLCKD group (p = 0.028) at week 8, whereas no significant differences among groups were observed at week 12 (Table 6 and Fig. 4).

**Table 6:** Adherence to diet at week 8 and 12

	ALL	VLCKD	HBD	p-value
Adherence to diet week 8	11.79 ± 2.17	12.4 ± 1.35	10.94 ± 2.8	0.028
Adherence to diet week 12	10.68 ± 2.42	11.2 ± 2.38	9.8 ± 2.3	0.080

#### Blood tests

**Fig. 5:**
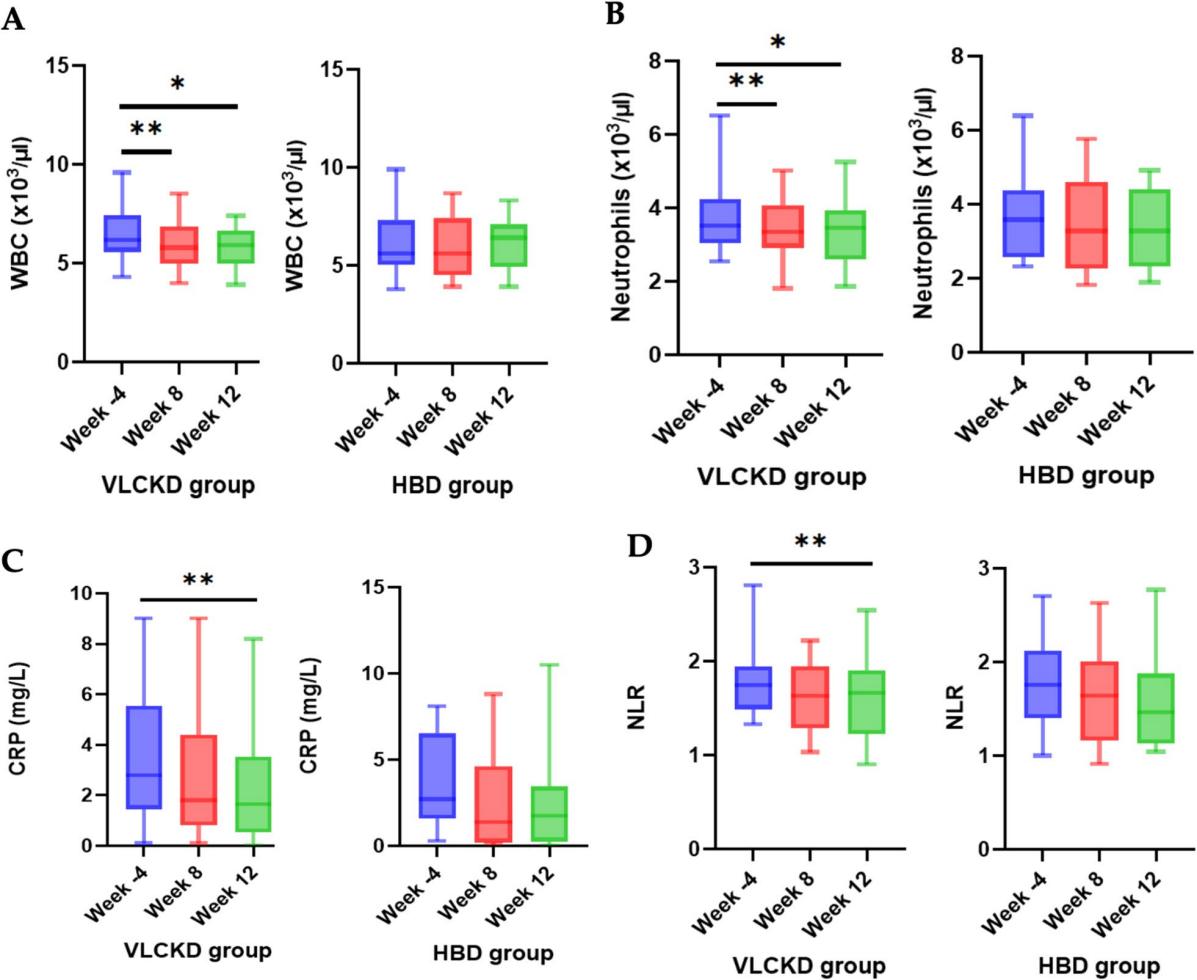
Box blot of mean change in WBC (**A**), Neutrophils (**B**), CRP (**C**), NLR (**D**), at week 4, week 8 and week 12 in VLCKD group and HBD group

**Fig. 6:**
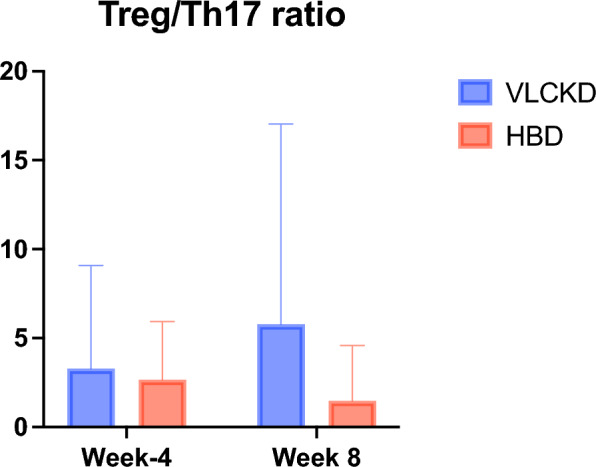
Changes in Treg/Th17 ratio at week 4 and week 8 in VLCKD group and HBD group

No significant variation in renal function (plasma creatinine and uric acid) were observed in subjects that followed VLCKD or HBD. GGT serum leves were significantly decreased in VLCKD group during the study. On the other hand, a significant reduction of ALT was found in HBD group, confirming the safety of both nutritional protocols. Importantly, white blood cell (WBC), platelets (PLT), neutrophils and monocytes were significantly reduced in the VLCKD group at week 8 (p = 0.001, p = 0.004, p = 0.002, p < 0.001, respectively) and week 12 (p < 0.05) (Fig. 5, Tables 7 and 8). Both dietary patterns (VLCKD and HBD) did not alter CRP, NLR and PLR at week 8 (Fig. 5); accordingly, lymphocytes subpopulations were not significantly altered in their frequencies in both study groups at week 8. Nonetheless, when we focused on the ratio between regulatory T cells (identified by the expression of CD25 and FoxP3, and by the lack of expression of CD127 on CD4 + T cells) and proinflammatory Th17 cells (CD4 + cells expressiong RORγt), we observed a trend of increase in Treg/Th17 ratio at V5 compared to V1 in VLCKD group, while this ratio was decreased in HBD group **(**Fig. 6). Although these variations did not reach statistical significance, they were suggestive of a general antiinflammatory effect of the VLCKD treatment. Along with this, we observed that CRP and NLR were significantly reduced at week 12 (p < 0.05) only in VLCKD group (Fig. 5, Table 8), confirming the well-known anti-inflammatory effect of VLCKD.

**Table 7:** Biochemical parameters (mean ± SD) at enrolling visit (week -4) and week 8 in VLCKD and HBD group

	VLCKsD	HBD	F, p-value
WBC			
* Week -4*	6.60 ± 1.41	6.22 ± 6.01	F = 1.544, 0.221
* Week 8*	5.93 ± 1.25	6.01 ± 1.58	
* Δ*	− 0.67 ± 1.00* p = 0.001	− 0.21 ± 1.48	
Hematocrit			
* Week -4*	42.3 ± 8.97	41.54 ± 3.43	F = 0.053, 0.818
* Week 8*	41.47 ± 2.46	41.27 ± 4.06	
* Δ*	− 0.84 ± 9.69	-0.26 ± 2.67	
PLT			
* Week -4*	278.54 ± 75.38	242.37 ± 62.41	F = 0.018, 0.895
* Week 8*	251.03 ± 65.96	237.31 ± 58.96	
* Δ*	− 27.5 ± 46.23* p = 0.004	− 5.06 ± 16.60	
Neutrophils			
* Week -4*	3.88 ± 1.04	3.62 ± 1.21	F = 0.728, 0.398
* Week 8*	3.40 ± 0.87	3.38 ± 1.19	
* Δ*	-0.48 ± 0.73* p = 0.002	− 0.24 ± 1.15	
Lymphocytes			
* Week -4*	2.11 ± 0.44	2.08 ± 0.46	F = 1.096, 0.301
* Week 8*	2.03 ± 0.44	2.11 ± 0.41	
* Δ*	− 0.08 ± 0.36	0.03 ± 0.33	
Monocytes			
* Week -4*	0.39 ± 0.14	0.35 ± 0.11	F = 2.701, 0.108
* Week 8*	0.31 ± 0.13	0.33 ± 0.10	
* Δ*	− 0.08 ± 0.10* p < 0.001	− 0.02 ± 0.13	
Glycemia			
* Week -4*	99.11 ± 8.64	96.00 ± 11.03	F = 1.041, 0.313
* Week 8*	96.78 ± 9.34	96.59 ± .7.92	
* Δ*	− 2.32 ± 8.38	0.59 ± 10.61	
AST			
* Week -4*	18.28 ± 6.54	19.76 ± 5.59	F = 1.048, 0.312
* Week 8*	19.11 ± 6.52	18.29 ± 6.60	
* Δ*	0.82 ± 7.14	− 1.12 ± 3.97	
ALT			
* Week -4*	20.64 ± 11.87	23.29 ± 13.64	F = 2.332, 0.134
* Week 8*	21.28 ± 12.97	18.29 ± 6.60	
* Δ*	0.64 ± 13.60	− 5.00 ± 8.71* p = 0.031	
GGT			
* Week -4*	22.50 ± 13.22	19.00 ± 7.66	F = 1.475, 0.231
* Week 8*	18.28 ± 12.08	17.23 ± 6.01	
* Δ*	− 4.21 ± 7.29* p = 0.005	− 1.77 ± 5.08	
Creatinine			
* Week -4*	0.69 ± 0.11	0.72 ± 0.12	F = 0.943, 0.337
* Week 8*	0.69 ± 0.09	0.74 ± 0.13	
* Δ*	− 0.003 ± 0.12	0.03 ± 0.08	
Uric acid			
* Week -4*	4.32 ± 1.35	4.52 ± 0.80	F = 0.443, 0.509
* Week 8*	4.50 ± 1.14	4.50 ± 0.77	
* Δ*	0.17 ± 1.16	− 0.01 ± 0.41	
CRP			
* Week -4*	4.91 ± 5.89	3.63 ± 2.80	F = 1.090, 0.302
* Week -8*	3.56 ± 4.60	4.39 ± 8.94	
* Δ*	− 1.34 ± 3.78	0.75 ± 9.53	
NLR			
* Week -4*	1.87 ± 0.56	1.75 ± 0.47	F = 0.002, 0.964
* Week 8*	1.71 ± 0.51	1.59 ± 0.47	
* Δ*	− 0.17 ± 0.43	− 0.16 ± 0.48	
PLR			
* Week -4*	136.74 ± 43.67	120.97 ± 38.23	F = 0.158, 0.693
* Week 8*	128.18 ± 41.11	116.15 ± 35.16	
* Δ*	-8.55 ± 33.62	-4.82 ± 21.92	
CD3 + T lymphocytes			
* Week -4*	62.04 ± 13.65	63.80 ± 10.20	F = 0.000, 0.992
* Week 8*	66.83 ± 12.70	68.53 ± 10.91	
* Δ*	4.79 ± 18.57	4.73 ± 9.89	
CD4 + T lymphocytes			
* Week -4*	60.00 ± 11.91	64.60 ± 13.94	F = 0.224, 0.639
* Week 8*	63.04 ± 15.90	64.87 ± 13.03	
* Δ*	3.0419.37	0.27 ± 14.90	
CD8 + T lymphocytes			
* Week -4*	29.95 ± 10.36	26.20 ± 10.52	F = 1.083, 0.305
* Week 8*	27.58 ± 12.61	28.47 ± 10.68	
* Δ*	− 2.37 ± 14.39	2.26 ± 12.03	
CD19 + B lymphocytes			
* Week -4*	18.77 ± 10.13	16.87 ± 9.98	F = 0.020, 0.888
* Week 8*	15.02 ± 12.16	12.47 ± 8.88	
* Δ*	− 3.75 ± 15.27	− 4.40 ± 11.53	
CD3- CD16 + CD56 + NK cells			
* Week -4*	15.80 ± 10.94	15.73 ± 7.33	F = 0.758, 0.390
* Week 8*	13.94 ± 7.47	17.67 ± 10.22	
* Δ*	− 1.86 ± 14.15	1.93 ± 11.83	
Naïve CD4 + T lymphocytes			
* Week -4*	22.20 ± 14.24	20.0 ± 9.21	F = 0.012, 0.912
* Week 8*	26.45 ± 14.21	23.60 ± 11.47	
* Δ*	4.24 ± 18.69	3.60 ± 15.65	
Naïve CD8 + T lymphocytes			
* Week -4*	30.30 ± 15.66	25.13 ± 13.67	F = 0.478, 0.494
* Week 8*	30.77 ± 14.21	30.62 ± 13.23	
* Δ*	0.47 ± 22.52	5.49 ± 21.24	
Regulatory T cells			
* Week -4*	1.01 ± 1.20	1.08 ± 1.41	F = 1.891, 0.178
* Week 8*	1.12 ± 1.20	0.40 ± 0.50	
* Δ*	0.10 ± 1.75	− 0.68 ± 1.59	
Th17 lymphocytes			
* Week -4*	0.32 ± 0.31	0.27 ± 0.24	F = 2.262, 0.150
* Week 8*	0.22 ± 0.21	0.50 ± 0.64	
* Δ*	− 0.10 ± 0.41	0.23 ± 0.55

**Table 8:** Biochemical parameters (mean ± SD) at enrolling visit (week -4) and week 12 in VLCKD and HBD group

	**VLCKD**	**HBD**	**F, p-value**
WBC			
Week -4	6.50 ± 1.43	6.08 ± 1.69	F = 2.042, 0.161
Week 12	5.74 ± 1.02	6.08 ± 1.44	
Δ	− 0.76 ± 1.50* **p < 0.05**	0.00 ± 1.82	
Hematocrit			
Week -4	42.91 ± 9.26	41.14 ± 3.67	F = 0.069, P = 0.794
Week 12	40.81 ± 2.28	39.75 ± 4.41	
Δ	− 2.09 ± 10.01	− 1.39 ± 3.09	
PLT			
Week -4	268.96 ± 62.51	244.73 ± 61.21	F = 1.796, p = 0.188
Week 12	245.28 ± 67.49	245.93 ± 83.25	
Δ	− 23.68 ± 54.96* **p < 0.05**	1.20 ± 59.95	
Neutrophils			
Week -4	3.84 ± 1.04	3.58 ± 1.25	F = 1.414, p = 0.242
Week 12	3.33 ± 0.86	3.52 ± 1.14	
Δ	− 0.51 ± 0.94* **p < 0.05**	− 0.06 ± 1.45	
Lymphocytes			
Week -4	2.07 ± 0.41	2.00 ± 0.52	F = 0.235, p = 0.631
Week 12	2.07 ± 0.43	2.07 ± 0.50	
Δ	0.002 ± 0.42	0.07 ± 0.41	
Monocytes			
Week -4	0.38 ± 0.13	0.33 ± 0.12	F = 1.490,p = 0.230
Week 12	0.30 ± 0.09	0.33 ± 0.15	
Δ	− 0.07 ± 0.12* **p < 0.05**	− 0.01 ± 0.18	
Glycemia			
Week -4	98.81 ± 8.83	97.87 ± 10.11	F = 4.858**,p = 0.033**
Week 12	93.73 ± 15.73	103.20 ± 14.25	
Δ	− 5.07 ± 14.14	5.33 ± 15.29	
AST			
Week -4	18.76 ± 6.78	20.27 ± 5.92	F = 4.275,**p = 0.046**
Week 12	18.36 ± 5.16	15.93 ± 3.01	
Δ	− 0.40 ± 5.95	− 4.33 ± 5.60* **p < 0.05**	
ALT			
Week -4	21.48 ± 12.27	24.80 ± 14.02	F = 2.499, p = 0.122
Week 12	18.60 ± 10.05	15.80 ± 6.32	
Δ	− 2.88 ± 12.07	− 9.0 ± 11.46*	
GGT			
Week -4	22.00 ± 12.14	20.67 ± 8.96	F = 0.428, p = 0.517
Week 12	18.16 ± 10.25	15.60 ± 4.76	
Δ	− 3.84 ± 5.65* **p < 0.05**	− 5.07 ± 5.89* **p < 0.05**	
Creatinine			
Week -4	0.72 ± 0.11	0.69 ± 0.11	F = 0.004, p = 0.949
Week 12	0.72 ± 0.10	0.72 ± 0.11	
Δ	0.029 ± 0.12	0.027 ± 0.10	
Uric acid			
Week -4	4.31 ± 1.38	4.41 ± 0.77	F = 0.214, p = 0.647
Week 12	4.37 ± 0.95	4.32 ± 0.94	
Δ	0.05 ± 1.03	− 0.09 ± 0.68	
CRP			
Week -4	4.32 ± 5.22	3.47 ± 2.54	F = 2.026, p = 0.163
Week 12	2.35 ± 2.33	3.96 ± 6.35	
Δ	− 1.97 ± 4.13* **p < 0.05**	0.49 ± 6.71	
NLR			
Week -4	1.82 ± 0.43	1.81 ± 0.48	F = 1.108, p = 0.299
Week 12	1.59 ± 0.41	1.87 ± 1.25	
Δ	− 0.22 ± 0.48* **p < 0.05**	0.059 ± 1.17	
PLR			
Week -4	131.61 ± 38.60	129.20 ± 43.10	F = 1.624, p = 0.211
Week 12	121.11 ± 28.58	130.44 ± 50.27	
Δ	− 10.46 ± 29.04	1.24 ± 25.93	
CD3 + T lymphocytes			
Week -4	64.29 ± 12.77	63.57 ± 10.59	F = 0.772, p = 0.386
Week 12	66.47 ± 16.38	70.50 ± 9.06	
Δ	2.17 ± 16.79	6.92 ± 13.79	
CD4 + T lymphocytes			
Week -4	58.76 ± 12.18	62.71 ± 14.71	F = 0.040, p = 0.843
Week 12	62.70 ± 13.63	67.71 ± 12.49	
Δ	3.93 ± 17.02	5.00 ± 12.61	
CD8 + T lymphocytes			
Week -4	30.76 ± 10.56	26.78 ± 11.50	F = 0.018, p = 0.894
Week 12	28.52 ± 12.27	25.21 ± 9.24	
Δ	− 2.23 ± 14.26	− 1.57 ± 14.20	
CD19 + B lymphocytes			
Week -4	18.39 ± 10.14	15.78 ± 10.51	F = 0.092, p = 0.764
Week 12	17.33 ± 9.69	16.14 ± 6.92	
Δ	− 1.06 ± 15.12	0.36 ± 10.77	
CD3- CD16 + CD56 + NK cells			
Week -4	13.90 ± 8.26	15.78 ± 7.61	F = 0.267, p = 0.609
Week 12	12.67 ± 8.83	12.43 ± 9.56	
Δ	− 1.22 ± 9.61	− 3.36 ± 14.80	
Naïve CD4 + T lymphocytes			
Week -4	22.61 ± 14.51	21.86 ± 10.09	F = 0.103, p = 0.750
Week 12	23.52 ± 13.65	20.79 ± 9.91	
Δ	0.90 ± 20.01	− 1.07 ± 13.88	
Naïve CD8 + T lymphocytes			
Week -4	28.34 ± 13.20	27.64 ± 13.44	F = 0.405, p = 0.529
Week 12	32.52 ± 15.24	27.50 ± 16.47	
Δ	4.18 ± 20.69	− 0.14 ± 18.00	
Regulatory T cells			
Week -4	0.92 ± 0.98	1.23 ± 1.46	F = 0.007, p = 0.932
Week 12	0.91 ± 1.50	1.29 ± 1.62	
Δ	− 0.005 ± 2.01	0.06 ± 2.35	
Th17 lymphocytes			
Week -4	0.45 ± 0.52	0.35 ± 0.31	F = 1.415, p = 0.279
Week 12	0.22 ± 0.17	0.95 ± 1.40	
Δ	− 0.22 ± 0.66	0.60 ± 1.23

At week 8 blood ketone levels was 0.86 ± 0.56 mmol/L, confirming the efficacy of VLCKD as well as patients adherence (the threshold value for nutritional ketosis was set at 0.5 mmol/L) [34].

Importantly, the reduction in glucose levels at week 12 was significantly higher in VLCKD group compared to HBD group (p = 0.003) (Table 8), in accordance with previous studies demonstrating the favourable effects of VLCKD on glucose control in the long term [40].

**Fig. 7:**
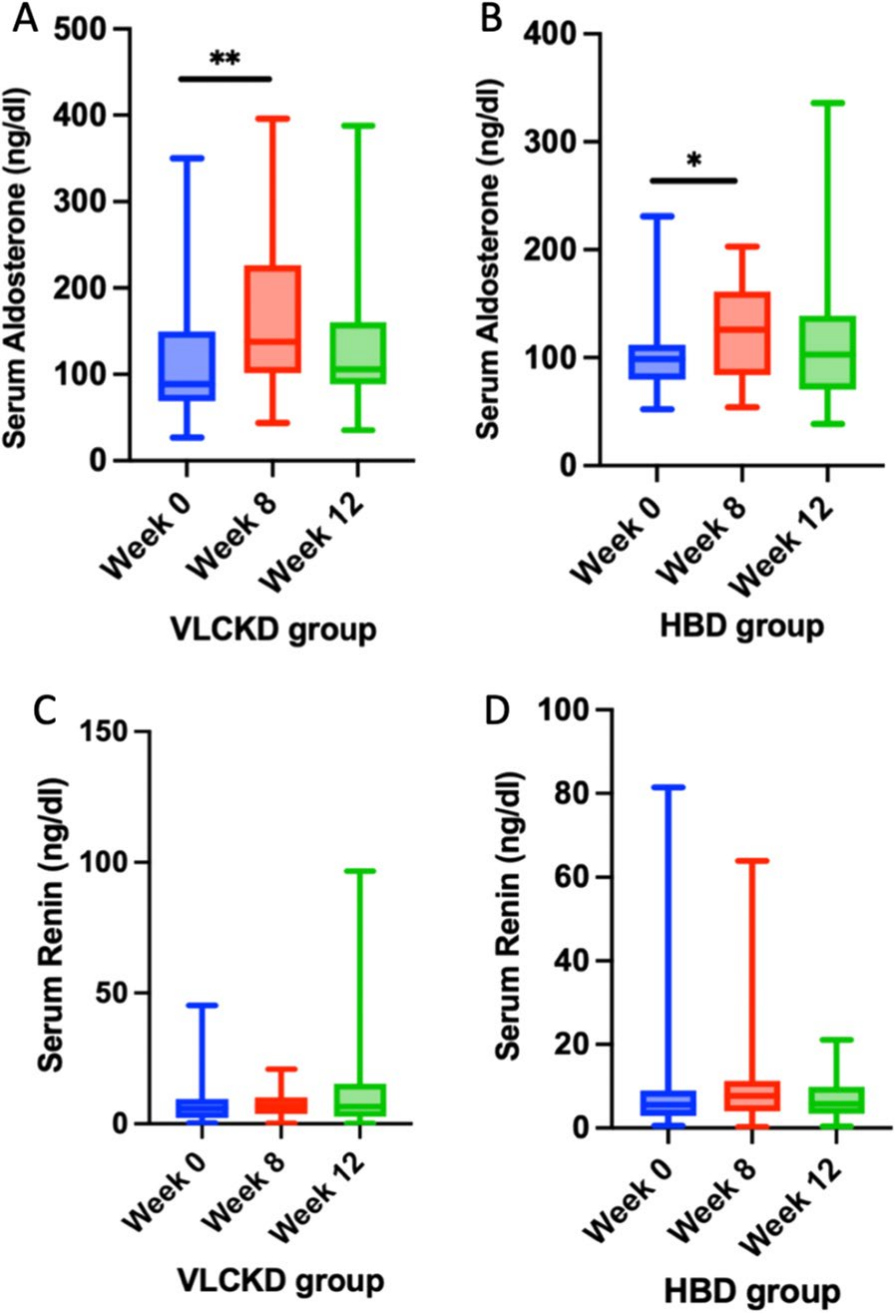
Box blot of mean change in serum aldosterone at baseline (week 0), week 8 and week 12 in VLCKD group (**A**) and HBD group (**B**). Box blot of mean change in serum renin at baseline (week 0), week 8 and week 12 in VLCKD group (**C**) and HBD group (**D**)

Aldosterone plasma level were significantly increased in both groups at week 8 (p = 0.003 in VLCKD group, p = 0.021 in HBD group—Fig. 7), with a major extent in VLCKD group, confirming recently published data [41], but not at week 12 (Table 9). However, sodium, potassium and direct renin plasma levels were never altered throughout the study in both dietary groups (Table 9), neither at week 8, nor at week 12.

**Table 9:** Electrolyte and hormonal profile at enrolling visit (week -4), week 8 and 12 in VLCKD and HBD group

Parameters		VLCKD	HBD	F, p-value		VLCKD	HBD	F, p-value
Sodium	*Week -4*	140.32 ± 2.83	140.35 ± 2.62	F = 0.586, 0.448	*Week -4*	139.80 ± 2.12	140.00 ± 2.56	F = 0.013, p = 0.909
	*Week 8*	140.29 ± 2.01	139.47 ± 2.23		*Week 12*	139.44 ± 1.38	139.73 ± 1.87	
	*Δ*	− 0.03 ± 3.61	− 0.88 ± 3.56		*Δ*	− 0.36 ± 2.69	− 0.27 ± 2.12	
Potassium	*Week -4*	4.68 ± 0.55	4.60 ± 0.43	F = 0.212, p = 0.648	*Week -4*	4.64 ± 0.55	4.61 ± 0.45	F = 0.212, p = 0.648
	*Week 8*	4.63 ± 0.59	4.41 ± 0.34		*Week 12*	4.84 ± 0.63	4.68 ± 0.72	
	*Δ*	− 0.05 ± 0.88	− 0.19 ± 0.52		*Δ*	0.20 ± 0.88	0.073 ± 0.78	
Aldosterone	*Week -4*	105.45 ± 67.71	93.78 ± 23.64	F = 1.152, 0.289	*Week -4*	99.07 ± 70.63	103.83 ± 38.52	F = 0.469, p = 0.498
	*Week 8*	161.50 ± 85.24	124.18 ± 46.67		*Week 12*	131.49 ± 78.75	115.69 ± 72.63	
	*Δ*	56.04 ± 90.48* p = 0.003	30.40 ± 49.09* p = 0.021		*Δ*	32.42 ± 94.45	11.86 ± 85.48	
Renin	*Week -4*	8.27 ± 9.34	11.60 ± 19.72	F = 0.008, 0.928	*Week -4*	7.97 ± 9.68	9.03 ± 9.75	F = 1.059, p = 0.310
	*Week 8*	7.42 ± 5.00	10.53 ± 14.35		*Week 12*	12.77 ± 19.71	7.66 ± 6.35	
	*Δ*	− 0.84 ± 8.07	− 1.07 ± 7.94		*Δ*	4.80 ± 22.58	− 1.37 ± 6.29	

Figure 7 shows mean change in serum aldosterone and renin at baseline (week 0), week 8 and week 12 in VLCKD group and HBD group.

#### Side effects

No serious side effects have been reported during the study in the two groups. In VLCKD group, 12 patients (41.4%) reported side effects, in particular 2 patients (16.6%) reported muscle cramps, 4 patients (33.3%) weakness, 1 patient (8.3%) hypotension, 3 patients (25%) constipation and 2 patients (16.6%) unintended weight loss.

4 patients (13.8%) showed poor compliance with VLCKD and decided to stop ketogenic diet referring dissatisfaction with weight-loss (25%), intolerance to carbohydrate restriction or craving for carbohydrates (25%), poor dietary variability (25%), the persistence of some residual migraine attacks despite the diet (25%). Remarkably, a relevant part of the clinical trial was performed during COVID pandemic.

One patient discontinued clinical trial because she tested positive for pregnancy.

14 patients (50%) of HBD group stopped clinical trial; the reasons that most frequently lead patients to reduce compliance or decide to stop with the HBD were: dissatisfaction with weight-loss (28.5%), poor time to prepare and organize meals (35.7%), changing family rituals and habits (14.3%) and lack of determination (21.4%).

## Discussion

This randomized controlled study brings new evidence of the efficacy of VLCKD in the management of high frequency episodic migraine in patients with overweight and obesity [42, 43].

Importantly, monthly migraine days (MMD) were significantly reduced by VLCKD, as compared with the control diet, at all time points during the study. At week 8, a significant difference in MMDs emerged between the two dietary groups (VLCKD − 6.4 ± 4.8 vs HBD − 2.2 ± 5.0, p = 0.008). A further increased mean reduction in MMD was observed in the VLCKD group at week 12, well beyond the end of ketosis phase, as well as at week 24, when all patients were on same dietary regimen (HBD). These results are even more interesting when considering that patients allocated to VLCKD had a more complex migraine picture compared to those allocated to HBD, documented by significantly higher baseline MMDs. Notably, weight loss and visceral fat reduction were also greater in VLCKD than HBD and persisted after ketosis cessation.

Concomitantly, VLCKD induced a significantly higher weight loss and visceral fat reduction (Fig. 3), compared to HBD, persisting at all time point during the study. Diet adherence was higher in VLCKD group (Fig. 4), probably due to ketone bodies anorexigenic effects and higher efficacy on reduction of body weight and migraine symptoms. The dual favorable action of VLCKD on migraine frequency and weight reduction has important implications on migraine course, because both HFEM and overweight/obesity represent independent risk factors for migraine chronicization [6, 7].

The therapeutic effect of ketone bodies in migraine relies on multiple mechanisms. Migraine brain is hyperexcitable and hypometabolic. Hence, ketone bodies represent a strategic and more efficacious metabolic fuel source, able to counteract defective neuronal glucose metabolism and to restore brain energy production in patients with migraine [14]. However, the persistency of VLCKD therapeutic gain over HBD, well beyond the phase of nutritional ketosis, suggests that VLCKD benefits in migraine prevention are not simply due to an improved energy metabolism, and probably include favorable effects on neuronal excitability and inflammation. KD is an established non pharmacologic treatment of epilepsy [44] and has been considered “*the most notable example of a dietary treatment with proven efficacy against a neurological condition*” [45]. The efficacy of KD in the management of different acute and chronic neurological diseases [46–48] relies on several mechanisms including reduction of neuronal excitability, neuro-inflammation and reactive oxygen species (ROS) production, restoration of neuronal myelin sheath, mitochondria formation and regeneration, reduction in glucose and insulin concentrations and amyloid plaques formation, and influence on intestinal microbiota composition, dopamine production and stimulation of glutamine conversion into GABA [19].

Also quality of life was improved during VLCKD treatment, particularly in several aspects of daily life, as general health (5.7 ± 11.7, p = 0.023), physical activity (10.6 ± 18.5, p = 0.009) and social relationship (12.2 ± 22.7, p = 0.013). When compared with HBD group, adherence to diet was higher in VLCKD group until week 8 (Fig. 4), suggesting that VLCKD represents a dietary model facilitating the patient to a better compliance and less cheat meal.

A hallmark of VLCKD is represented by a prolonged mild ketosis state, which allows an acceptable tolerance to the nutritional regimen due to its anorexigenic actions, and to its favourable effects on general well-being, and only transient mild side effects, [14, 49].

The present study documents anti-inflammatory effects in patients treated with VLCKD but not in those receiving HBD. In the former, we observed a reduction in white blood cells count and neutrophils at all study time points, in keeping with previous reports [50]. The effect on neutrophils is likely to account for the reduction in NLR ratio, a reliable marker of inflammation, observed at week 12. Importantly, CRP plasma levels were also significantly reduced at all time points in VLCKD group, confirming the anti-inflammatory effects of this dietary protocol.

Lymphocytes subpopulations levels were not significantly altered by VLCKD, confirming the preservation of specific immune responses after this dietary regimen. However, Treg/Th17 ratio was increased in VLCKD group, and reduced in HBD group, even if these effects did not reach statistical significance (Fig. 6). Treg and Th17 are strictly interconnected, as their development relies on shared cytokines (i.e. TGFβ), and the balance between the two populations is driven by the levels of cytokines in the microenvirornment [51]. The Treg/Th17 balance is particularly relevant in mantaining a proinflammatory or anti-inflammatory status [52]. Our observation is in line with previously described reduction of the Th17 population in patients treated with VLCKD [53].

We then wanted to explore the impact of both dietary regimens on plasma aldosterone, renin and salt balance, given the strong implication of RAAS in the pathophysiology of both migraine and obesity. Interestingly, VLCKD significantly increased aldosterone plasma level at week 8 (p = 0.003, Fig. 7), in line with recently published data [41]; such effect was not observed at week 12. A mild increase in plasma aldosterone was also observed in HBD group, although at a lower extent (p = 0.021, Fig. 7), compared to VLCKD group. Sodium, potassium and direct renin plasma levels were never altered throughout the study in both dietary groups (Table 9), neither at week 8, nor at week 12, suggesting a renin independent RAAS activation, potentially due to direct effects of ketone bodies on aldosterone production by the adrenal gland. In this context, increased plasma aldosterone levels did not contribute to alter electrolyte balance, nor inflammatory markers and blood pressure control, and probably simply reflected a rapid RAAS adaptation to a dietary regimen favouring salt and water loss.

A strenght of this study is represented by the multidisciplinary team (including neurologists, nutritionists, psychologists and endocrinologists) who followed patients during the clinical trial, which increased adherence to the study. Importantly, the study was characterised by a long term follow up, since patients were followed for at least 24 weeks, well beyond the end of the ketosis phase, and included a special population of patients, presenting a high risk of chronicization. Finally, we evaluated the impact of nutritional treatments on the response of the immune system (with particular regard to inflammatory and regulatory T cells), and RAAS adaptation to dietary regimens and weight loss.

This study has several limitations: first of all the number of subjects enrolled is small, with several treatement discontinuations, mostly due to COVID pandemic. Moreover, our patients’ population included a small proportion of male patients (5.1%), in line with migraine epidemiology [5]. Second, baseline MMDs were higher at baseline among VLCKD patients, as compared to HBD group. However, such randomization bias adds more strength to the results obtained on MMD reduction in patients allocated to the VLCKD, who presented a definitely worse clinical condition. Finally, this trial has been conducted during COVID-19 pandemic; this particular moment may have negatively impacted on people adherence to dietary pattern and final results.

## Conclusions

Findings from the current trial brings new evidence to the clinical efficacy of ketogenic diets and strongly suggests that VLCKD represents an effective treatment or co-adjuvant therapy, in combination with pharmacological approaches, for the management of HFEM in patients with overweight or obesity, who are at higher risk of chronicization, compared to general migraine population. VLCKD might represent a valuable treatment tool also as add-on therapy in patients with unsatisfactory response to conventional migraine pharmacological prophylaxis. Further, as obesity is a negative predictor of responsiveness to monoclonal antibodies (mAbs) targeting the calcitonin gene-related peptide (CGRP) in patients with chronic migraine, VLCKD could be recommended in patients with elevated BMI values not responding to antiCGRP mAbs [12].

However, future studies are deemed necessary to confirm our findings in larger, long term, multicenter clinical trials.

## Data Availability

The data sets generated and/or analyzed during the current study are not publicly available but are available from the corresponding author on reasonable request.

## Abbreviations

T2DM: Type 2 diabetes mellitus
VLCKD: Very low-calorie ketogenic diet
WC: Waist circumference
HC: Hips circumference
BP: Blood pressure
LDL: Low density lipoproteins
HDL: High density lipoproteins
TG: Triglycerides
KD: Ketogenic diet
BMI: Body mass index
WHR: Waist to hip ratio
GFR: Glomerular filtration rate
HBD: Hypocaloric balanced diet
HFEM: High-frequency episodic migraine
MMD: Monthly migraine days
RAAS: Renin–angiotensin–aldosterone system
VAS: Visual analogue scale
HIT-6: Headache impact test-6
SF-36: Short form health survey-36
